# Dealing with diabetes and pregnancy following bariatric surgery: a double-edged sword?

**DOI:** 10.1590/2359-3997000000181

**Published:** 2016-08-01

**Authors:** Marcio C. Mancini

**Affiliations:** 1 Hospital das Clínicas Faculdade de Medicina Universidade de São Paulo São Paulo SP Brasil Grupo de Obesidade e Síndrome Metabólica da Disciplina de Endocrinologia e Metabologia do Hospital das Clínicas da Faculdade de Medicina da Universidade de São Paulo (HC-FMUSP), São Paulo, SP, Brasil

Bariatric surgery offers substantial and sustained weight loss for most patients, with diabetes improvement or remission and also reduction in weight-related comorbidities in patients with a BMI of 35 kg/m^2^ or more. The same benefits have not yet been established for patients with a BMI of less than 35 kg/m^2^, since there is still limited evidence based in very few studies investigating less than one hundred patients with class I obesity. Moreover, in larger studies involving patients with higher baseline BMI such as the Swedish Obese Subjects Study, the degree of weight loss has been significantly associated with glycemic improvement, independently of the surgical procedure when stratified by weight. In spite of this, delegates of International Bariatric Surgery and Diabetes Organizations during the 2^nd^ Diabetes Surgery Summit in London recently approved a joint statement where surgery should be considered (consider should not have the same strength of a recommendation) for patients with type 2 diabetes and BMI between 30.0 and 34.9 kg/m^2^ if hyperglycemia is inadequately controlled despite optimal treatment with either oral or injectable medications, including insulin; and these BMI thresholds should be reduced by 2.5 kg/m^2^ for Asian patients (in spite of short duration of diabetes, good glycemic control and absence of insulin therapy be preoperative predictive factors of diabetes remission) (1). Recently, some cardiovascular safety trials with diabetes medications have demonstrated that these drugs offer cardiovascular protection (2); more data on hard outcomes are required to better assess not only the efficacy, but mainly the safety in very large series before endorsing the widespread indication of bariatric surgery in the subpopulation of type 2 diabetics with a BMI below 35 kg/m^2^. A significant proportion of individuals undergoing bariatric surgery experiment weight regain, residual diabetes or diabetes relapse (i.e., around one third of initial remitters over five years do not achieve remission or have diabetes recurrence), requiring to cope with the residual diabetes and emerging obesity (2,3) and also to deal with lifelong nutritional deficiencies and other potential long-term complications, such as vomiting, adhesions, strictures, gallstones, hernias, drinking problems, and small-bowel obstruction (4,5). In the scope under discussion, it is essential that the diagnosis of diabetes in patients undergoing bariatric surgery be reliable and safe.

In this issue of *Archives of Endocrinology and Metabolism*, a study examined the adverse effects of the oral glucose tolerance test (OGTT) in 128 patients who underwent bariatric surgery, of which more than 90% were women and nearly 30% pregnant. Around two thirds of patients experienced one to four or more limiting adverse effects such as nausea, dizziness, weakness, diarrhea, tachycardia, sweating or hypoglycemia during the test. The main reasons for ordering an OGTT were presence of symptoms suggestive of hypoglycemia, pregnancy, type 2 diabetes prior to surgery, or weight gain (6). In effect, an OGTT, when performed in patients following bariatric surgery (mainly but not limited to Roux-en-Y gastric bypass) is linked to risks, and it is questionable for evaluating this cohort of patients (5).

In recent years, episodes of severe late postprandial hypoglycemia have been increasingly reported in gastric bypass patients. In the evaluation of these most frequently asymptomatic hypoglycemic events, fasting and post-prandial self-monitoring reflectance meter glucose determinations in the home setting, or fasting and post-prandial blood glucose concentration measurements can be requested occasionally and, if symptoms of hypoglycemia occur likewise (5,7,8). These patients could also be evaluated by ambulatory continuous measuring of interstitial fluid glucose systems (5,8) or a mixed meal test, preferably liquid (7).

The same approach has been considered valid in the evaluation of remission and relapse of type 2 diabetes mellitus, as well as measurement of glycated hemoglobin levels (5,8). Over the years, many different criteria of diabetes remission have been proposed based on maintaining fasting glucose and glycated hemoglobin levels below stricter or less strict thresholds for shorter or longer periods of time (*e.g*. 1, 2, 5 years postoperatively) (9). At the same time, a higher mean amplitude of glucose excursions (MAGE), a shorter time to the post-prandial peak of interstitial glucose occur (with maximum glucose levels above 200 mg/dL, but with normal fasting and 2-hour post meal levels in most patients with a diabetes background before surgery), and symptoms of hypoglycemia usually were more related to the speed of glucose decrease than to the glucose level achieved, since hypoglycemic episodes were not registered on all occasions. Being so, the early post-prandial hyperglycemia may underestimate the diagnosis of diabetes if glucose measurement is done 2-hours post-meal (10).

Furthermore, glucose variability is exaggerated after Roux-en-Y gastric bypass, combining typically high and early hyperglycemic peaks and rapid glucose decreases with symptoms mimicking hypoglycemia (but on occasion being true severe hypoglycemia) (10). There are high quality evidence criteria associating post-meal and post-challenging glucose hyperglycemia and macrovascular disease, retinopathy, increased carotid intima-media thickness, oxidative stress, inflammation and endothelial dysfunction, impaired cognitive function in elderly people with type 2 diabetes, as well as decreased myocardial blood flow, and increased risk of some types of cancer. A task force of the International Diabetes Federation strongly states that post-meal hyperglycemia is harmful and should be addressed. Treatments that aim post-meal plasma glucose reduce vascular events as long as hypoglycemia is avoided. Both non-pharmacologic, such as diets with a low glycemic load, and pharmacologic therapies that preferentially lower post-meal plasma glucose targeting post-meal plasma glucose below 140 mg/dL (7.8 mmol/l) (11).

Thus, instead of OGTT, a mixed meal test (7) and/or self-monitoring of blood glucose should be preferred for diabetes diagnosis of patients undergoing bariatric surgery since it is currently the most practical method for monitoring post meal glucose excursions, and also helps to register and to avoid hypoglycemia (11). There is evidence suggesting that employing only fasting blood glucose and glycated hemoglobin levels to diagnose diabetes in patients undergoing bariatric surgery leads to underestimation, since the glycated hemoglobin may be falsely minimized or normal in patients with higher MAGE (especially with concomitant both hypoglycemic and hyperglycemic events) (10). Although these parameters provide good specificity, the sensitivity is poor in the diagnosis of dysglycemia (12).

Nearly a third of patients studied were pregnant women who underwent bariatric surgery (6). Even if significant weight loss reduces the risk of gestational diabetes mellitus (GDM), most women remain overweight after bariatric surgery, and, thus, should be screened for GDM. The assessment can be accomplished between 24 and 28 weeks of gestation by fasting and post-prandial reflectance meter glucose determinations or blood glucose concentrations or also be evaluated by ambulatory continuous measuring of interstitial fluid glucose systems (5,8). Many women who were unable to get pregnant before surgery conceive after bariatric procedures, suggesting a beneficial influence on fertility outcomes. Studies that systematically monitor adherence and nutritional status are scarce, but women in reproductive age are particularly prone for anemia, and mineral and vitamin deficits following Roux-en-Y gastric bypass. Further, patients did not adequately follow the multivitamin prescription despite careful instructions. Folic acid is considered a marker of multivitamin intake and was below the reference level in around 10% of female patients. Only 36% (24/67) of female patients 8 years following gastric bypass had a reliable intake of multivitamin supplementation (13), increasing the possibility of vitamin deficiencies during pregnancy, thus posing risks to fetal development with long-lasting health consequences such as cognitive and motor impairment, neural tube defects, and maternal or maternal-fetal complications during pregnancy, including severe malnutrition requiring parenteral nutrition, and, hardly ever, death. Long-term outcomes for both the mother and her offspring are still needed (14).

Women who had undergone previous bariatric surgery (comprising more than two thousand singleton births to a mother with a history of gastric bypass, adjustable gastric band or vertical gastroplasty adjusted by maternal age, parity, early pregnancy body mass index and smoking status, educational level) have a higher rate of preterm birth than those who had not and are more likely to deliver a small-for-gestational-age infant compared with those who did not have the operation. It may be reasonable for the obstetrician to screen these pregnancies for the development of intrauterine growth restriction (15). It is not entirely clear whether this increases the risk to the fetus or has long-term consequences for his health in adulthood, but some studies suggest that maternal bariatric surgery increases the risk of metabolic disorders in the offspring. Female rats rendered obese on a high-calorie diet underwent either sleeve gastrectomy or sham surgery. Pups born to sleeve gastrectomy rats were born smaller and lighter than the offspring of control rats. Moreover, when maintained on a high fat diet after puberty, these adult offspring had a greater propensity to develop glucose intolerance, hypercholesterolemia, hepatic steatosis, and overweight or obesity than the control offspring of rats who underwent sham surgery (16). Many factors in the maternal environment can result in intrauterine growth restriction including calorie or macronutrient malnutrition, malabsorption, anemia, loss of maternal ghrelin producing cells (as a putative regulator of intrauterine growth and development, acting on GHS receptors in the placenta and offspring), thus suggesting that the maternal bariatric surgery could increase the risk of obesity, metabolic syndrome and diabetes in the next generation (14-16).

To conclude, using an OGTT in patients who underwent bariatric surgery in order to evaluate hypoglycemia, or to diagnosis of GDM, type 2 diabetes, or diabetes relapse undoubtedly, leads to rapid gastric emptying of the hypertonic glucose solution causing a dramatic shift of fluid from the intravascular component to the intestinal lumen with relative hypovolemia, increase in bowel contractility (consequences of excessive secretion of hormones vasoactive intestinal polypeptide, serotonin, and bradykinin) and, incomplete compensatory sympathetic activity, pivotal in the early dumping symptoms. The rapid small intestine transit of hyperosmolar glucose causes a massive release of the incretin hormones glucose-dependent insulinotropic polypeptide and glucagon-like peptide-1 (GLP-1) that stimulate an exaggerated insulin secretion causing reactive hypoglycemia (further aggravated by the inhibition of glucagon secretion by GLP-1) and the symptoms of late dumping symptoms (17). The evaluation of these patients by ambulatory continuous measuring of interstitial fluid glucose systems seems to be more sensitive in detecting hypoglycemia, hyperglycemia, for the diagnosis of diabetes, diabetes relapse, excessive glucose variability, and also GDM.
